# A systematic review of automated hyperpartisan news detection

**DOI:** 10.1371/journal.pone.0316989

**Published:** 2025-02-21

**Authors:** Michele Joshua Maggini, Davide Bassi, Paloma Piot, Gaël Dias, Pablo Gamallo Otero

**Affiliations:** 1 Centro Singular de Investigación en Tecnoloxías Intelixentes (CiTIUS), Universidade de Santiago de Compostela, Galicia, Spain; 2 IRLab, CITIC Research Centre, Universidade da Coruña, A Coruña, Galiza, Spain; 3 Université Caen Normandie, ENSICAEN, CNRS, Normandie Univ, GREYC UMR6072, F-14000 Caen, France; Universidad Diego Portales, CHILE

## Abstract

Hyperpartisan news consists of articles with strong biases that support specific political parties. The spread of such news increases polarization among readers, which threatens social unity and democratic stability. Automated tools can help identify hyperpartisan news in the daily flood of articles, offering a way to tackle these problems. With recent advances in machine learning and deep learning, there are now more methods available to address this issue. This literature review collects and organizes the different methods used in previous studies on hyperpartisan news detection. Using the PRISMA methodology, we reviewed and systematized approaches and datasets from 81 articles published from January 2015 to 2024. Our analysis includes several steps: differentiating hyperpartisan news detection from similar tasks, identifying text sources, labeling methods, and evaluating models. We found some key gaps: there is no clear definition of hyperpartisanship in Computer Science, and most datasets are in English, highlighting the need for more datasets in minority languages. Moreover, the tendency is that deep learning models perform better than traditional machine learning, but Large Language Models’ (LLMs) capacities in this domain have been limitedly studied. This paper is the first to systematically review hyperpartisan news detection, laying a solid groundwork for future research.

## Introduction

The foundation of democratic governments rests on the voting process conducted by citizens [1]. Political parties, in their quest for votes, heavily rely on news media to disseminate their messages during campaigns. While transparent information and active political participation are crucial for a healthy democracy, political entities increasingly employ hyperpartisan communication strategies. These tactics aim to discredit opposing factions and distort reality, potentially impacting how governments represent their constituents. Although hyperpartisan campaign methods may increase voter participation [2] and strengthen the connection between voting decisions and specific ideologies, they can have significant negative consequences. As [3]emonstrates, this communication style can highlight divisive tensions within society, complicating governance and potentially alienating citizens when opposing sides gain power. Consequently, hyperpartisanism poses a threat to the proper functioning of democracy [4] by polarizing and dividing the social fabric, reducing trust in governmental entities and mainstream news [5], and exacerbating tensions between governments and their oppositions [6].

The rise of alternative media outlets further amplifies these threats to democracy [7], as they often share polarizing content [8]. In the online sphere, hyperpartisanship proliferates through various channels social networks publishers’ websites. The dissemination of hyperpartisan news, characterized by highly polarized political and ideological content, capitalizes on the virality facilitated by platform algorithms [9]. While the term gained prominence during the 2016 U.S. election [10], there is no evidence suggesting that this specific event triggered a systemic hyper-polarization [11].

The digital realm has become a significant arena for political influence [12], affecting the entire infosphere [13]. The close relationship between hyperpartisanship and online interactions has led to increased attention on these manipulative forms of communication [14]. On the policy front, the EU Commission’s 2018 expert report [15] addressed related topics such as disinformation, defamation, hate speech, and incitement to violence. More recently, the European Parliament adopted the Digital Services Act (DSA) [16] in 2022, aiming to provide "a secure, predictable and trustworthy online environment" (Article 1. 1). In line with [9] and [17], we categorize hyperpartisan news under the broader umbrella of misinformation, closely related to fake news detection. Hyperpartisan news detection as a classification task is specifically related to the news domain and can focus on linguistic, semantic, and meta-data features. The objective is for an algorithm to predict a text’s political affiliation or determine if the content is hyperpartisan. The rising academic interest in hyperpartisan detection is testified by the high participation of 42 teams at task 4 of SemEval-2019 [18].

For this systematic review, we only considered automated text-based strategies applied to news articles. Manual detection of hyperpartisan news has been proposed. It mainly focuses on discourse analysis [19–21]. Despite its effectiveness, this approach does not scale with the daily news spreading. Hence, automated methods such as deep learning, social network analysis, or cross-methodologies like [22] are more effective. These approaches rely on different features, so that hyperpartisan news detection may be tackled adopting content, sources, and user-based data [23].

The article is organized as follows: the *Related Works* section covers the relevant surveys on similar topics, highlighting the main features and comparing their limitations with regards to our study; the *Methodology* section discusses the methodology adopted for this systematic review, including research questions, search strategy, criteria selection, and selection procedure; the section *Hyperpartisan news detection: description of the phenomenon* focuses on the definition of hyperpartisanship, highlighting its multi-task and cross-disciplinary nature. Afterward, we present the textual frames where hyperpartisan traits are traceable and the spectrum of methodologies used in different computational sub-fields. Then, we covered the diverse strategies and scales used to label hyperpartisanship. Section *Approaches for automatic hyperpartisan news detection* contains a global categorization and discussion of the most performant model in the papers screened and selected. We distinguished between the typology of the model, the results, the features and the approaches employed. Section *Datasets* is a descriptive overview of the datasets used in this domain: we collected the cited datasets and their features. Finally, section *Conclusions and future works* concludes the article by presenting the main findings of our literature review.

The main contributions of this study are:

- Comparing the different definitions of hyperpartisan news detection;
- Collecting and discussing the diverse approaches and algorithms used in the selected literature, specifically for the news domain;
- Reporting evaluation metrics, features and embeddings considered in the studies;
- Presenting the main findings, the engineering innovations and research designs;
- Collecting and analyzing 38 datasets used in the literature, focusing on English and less representated languages;
- Delineating prevailing research gaps and challenges in hyperpartisan news detection task.

## Related works

The current state of the literature lacks a systematic review specialized in automatic hyperpartisan news detection. While there are various relevant survey papers, they predominantly focus on fake news and bias detection tasks. For instance, [24] examined fake news detection while considering the relation between factuality and political bias of news sources without showing any dataset or discussing the methodologies. [25] started from a theorical introduction of the fake news phenomenon to then cover the technical methodologies considering different perspectives from content to style analysis. [26] compared manual and automated approaches to identify media bias, distinguishing several forms of bias occurring in the distinct steps of news production. Similarly, [27] investigated the application of deep learning algorithms in fake news detection, building upon a taxonomy proposed by [17], where hyperpartisan news detection overlapped with fake news detection. [28] covers the broad field of disinformation by designing a taxonomy without considering either automated approaches or the datasets used in the literature. Similarly, [29] analyzes the general phenomenon of media bias detection by describing its diverse manifestations (e.g., spin bias, ideology bias, coverage bias), distinguished the techniques to detect them and reported 17 datasets. Except for this last author, no particular attention was given to hyperpartisan news detection from the others.

## Methodology

In this section we will present and describe the methodology adopted to conduct this systematic review following [30]’s guidelines. The planning and execution phases of this study are detailed in the following subsections, while the results phases are discussed in section *Hyperpartisan news detection: description of the phenomenon*, section *Approaches for automatic hyperpartisan news detection* and section *Datasets*.

### Research Questions

The Research Questions (RQ) that motivated the need for this systematic review are the following:

RQ1 Does a categorization for hyperpartisan news detection methods exist?
RQ2 Is hyperpartisan news detection a stand-alone or over-lapping task?
RQ3 What are the proposed solutions using textual data?
RQ4 Does the task keep up with the new Natural Language Processing technologies like autoregressive models?
RQ5 What are the results of the models developed?
RQ6 What are the datasets used for this task? How are they structured? Have they been updated to cover the latest political global and regional trends?
RQ7 How can the current state of research on hyperpartisan detection be characterized in diverse languages and countries?

### Search strategy

**Fig 1:**
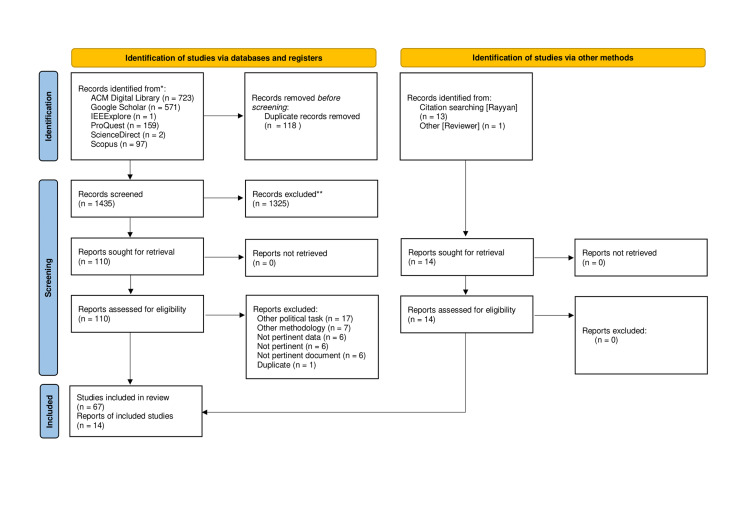
PRISMA Flow Diagram. The Flow Diagram illustrates the steps during document collection and evaluation. We skimmed more than 1553 papers and finally we selected a subset of 81.

We adopted the Preferred Reporting Items for Systematic Reviews and Meta-Analysis (PRISMA) guidelines [31], consisting of a checklist (http://www.prisma-statement.org/documents/PRISMA_2020_checklist.pdf) and a flow diagram Fig 1 to illustrate in a simplified and clear way the steps made. To retrieve papers, primary different academic online databases were used to overcome their respective limitations [32] in terms of topic coverage and papers available: ACM Digital Library, Google Scholar, Scopus, ProQuest, and IEEExplore. Our query archetype was: ((hyperpartisan OR “political bias” OR “hyper-partisan” OR partisanship OR hyperpartisanship OR “political polarization”) AND (news OR bias OR articles) AND (detection OR classification)). The first set of words contains the different homographs. We also searched in all subject fields, to capture as many semantically similar papers as possible, including potentially miscategorized papers. We selected the 2015-2024 timeframe to analyze trends before the term was coined, considering a period in which studies on this topic grew, and increasingly powerful models were employed.

For the purpose of obtaining pertinent papers related to our questions, the queries reported in Table 1 were a refined result of a structured process based on different steps introduced by [29]. Queries within each database were structured to match titles, abstracts, and keywords. We extended this pipeline, introducing the following step: “Network visualization and exploration”. It concerned the usage of the research software ResearchRabbit (https://researchrabbitapp.com) to exhaustively capture possibly omitted papers through the citation links structure.

- **Keywords domain extrapolation:** Initial reviews on similar topics helped us identify the keywords used in this domain. We noticed a lack of scientific agreement on writing “hyperpartisan”. To cover all these morphologically diverse forms (hyper-partisan, hyperpartisan, hyper-partisan), we included them in our queries, treating them as synonyms;
- **Iterative searches:** This process allowed us to select the most appropriate terms by comparing the results retrieved using different keywords combinations. We examined how much titles and abstracts related with the queries;
- **Verifying against established literature:** To ensure the efficiency of our search terms, we compared the results to a list of papers in the domain of hyperpartisan detection;
- **Network visualization and exploration:** To further validate our verification, we used ResearchRabbit, a tool to visualize the citation links between papers in the same collection. It suggested similar papers written by the same or different authors, highlighting stored publications in the user’s folder. This tool helped us in gauging the coherence of our results.

**Table 1:** Queries performed with advanced search for each database and the number of papers retrieved.

Database	Query	Docs
ACM Digital Library	[[[Title: hyperpartisan] OR [[Title: political polarization] AND [[Title: news] OR [Title: article]]]] AND [[Abstract: hyperpartisan] OR [Abstract: media bias] OR [Abstract: or news] OR [All: article]]] OR [All: and] OR [[[Keywords: hyperpartisan] OR [[Keywords: media bias] AND [[Keywords: news] OR [Keywords: article]]]] AND [[Title: hyperpartisan] OR [Title: hyper-partisan] OR [Title: partisan] OR [Title: political polarization] OR [[Title: media bias] AND [[Title: news] OR [Title: article]]]]] AND [E-Publication Date: (01/01/2015 TO 12/31/2024)]]	723
Google Scholar	(hyperpartisan OR hyper-partisan OR hyperpartisanship OR hyperpartisan ORpolarization) AND (news OR bias OR articles) AND (detection ORclassification)	1800
IEEE Xplore	(All Metadata:hyperpartisan OR All Metadata:hyper-partisan OR All Metadata:hyperpartisanship OR All Metadata:hyper-partisanship) AND (All Metadata:detection)	1
ProQuest	hyperpartisan news + filters	159
ScienceDirect	(hyperpartisan OR hyper-partisan OR political polarization OR media bias) AND NLP	2
Scopus	(hyperpartisan OR hyper-partisan OR partisanship OR hyperpartisanship OR political polarization) AND (news OR articles OR bias) AND (classification OR detection))	97

### Selection criteria

Before describing the screening process, we illustrate the criteria employed for the paper selection.

- Inclusion criteria
  - Papers primarily focused on automated hyperpartisan news detection;
  - Papers that used the related task (e.g. fake news detection) as a synonym of hyperpartisan news detection;
  - Publications from 2015 to 2024;
- Exclusion criteria
  - Exclusion of sources that either address the hyperpartisan news detection problem from a theoretical perspective, namely theory papers, or manual detection;
  - Studies discussing only related topics, such as fake news detection, stance detection, or political bias;
  - Findings that do not use news domain datasets as the main source for hyperpartisan news detection, i.e. social network analysis, comments analysis, and tweets detection-based approaches;
  - Literature reviews, books, thesis and posters.

### Screening and selection process

The following search strategy and procedures for study selection and analysis were used. The study selection, quality assessment of the included studies, and thematic analysis were performed by one author (PP). However, the procedures and findings were discussed by all authors, and potential disagreements were resolved by consensus.

To manage the screening and selection processes, we utilized *Rayyan* (https://www.rayyan.ai/) for its AI-powered capabilities, which allowed the two reviewers to conduct a blinded selection process, preventing any mutual influence. Specific eligibility criteria were established to ensure the reliability of the study. These criteria were applied independently by each reviewer to maintain objectivity and consistency. The criteria included: relevance to the predefined inclusion criteria, evaluation of models using both accuracy and F1 score, and comprehensive reporting of the dataset used. Only papers that met the criteria and were accepted by both reviewers were selected. In cases where there was disagreement, a third reviewer was consulted to assess the paper’s eligibility. The initial dataset consisted of 723 papers from ACM Digital Library (https://dl.acm.org/), 571 from Google Scholar (https://scholar.google.com/), 1 from ScienceDirect (https://www.sciencedirect.com/), 97 from Scopus (https://www.scopus.com/home.uri), 159 from ProQuest (https://www.proquest.com/index), and 1 from IEEE Xplorer (https://ieeexplore.ieee.org/Xplore/home.jsp). Notably, Google Scholar initially retrieved 1800 results, but we noted that, after the threshold of 500 results, it did not produce relevant documents. That led us to manually collect only the first 571 papers.

We conducted the entire selection process using Rayyan, as described in Fig 1. It automatically detected 118 duplicates. After manual checks, we removed them. Left with 1441 studies, screening titles and abstracts was the initial step. Following thorough evaluations, 67 papers were retained from a curated pool of 110, eliminating 43 papers that did not meet specific focus or dataset criteria.

**Fig 2:**
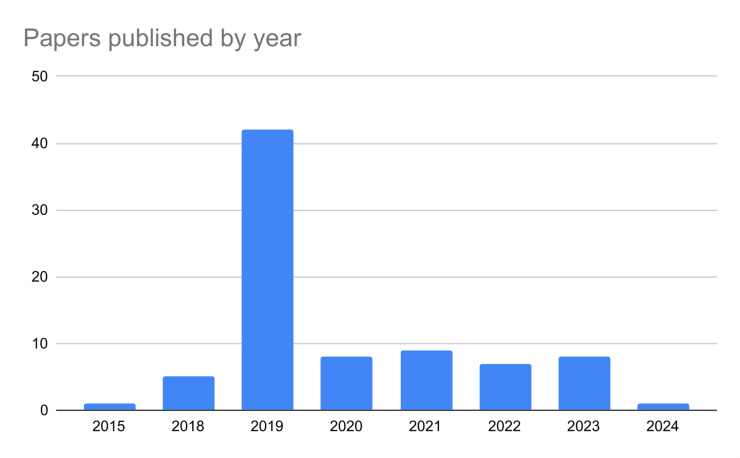
The bar chart illustrates the trend of the selected publications over time.

**Fig 3:**
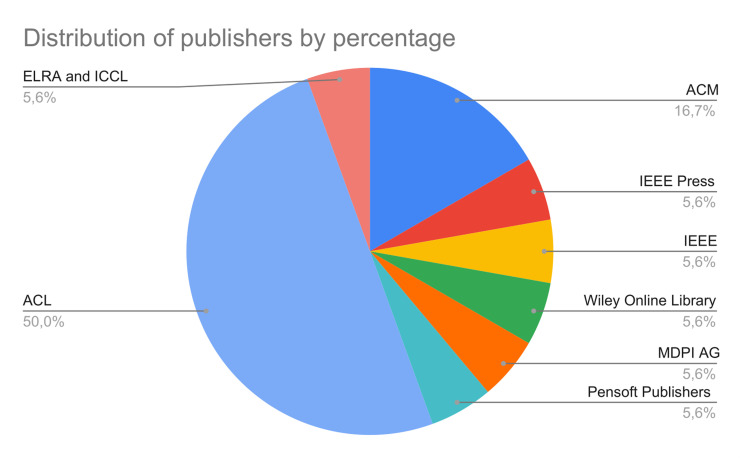
The pie chart shows the main publishers for the selected papers.

Additionally, to examine the cohesion and coherence of our references, we used ResearchRabbit to visualize the citation network, identifying two prominent clusters with centers in [33] and [18]. [18] is a key work from the SemEval initiative, set a foundational benchmark for detecting hyperpartisan in news articles, which informed our criteria for selecting relevant studies. This shared task saw the participation of 42 teams. They explored several approaches that future research will expand upon it. Moreover, the two datasets described in are important benchmarks for hyperpartisan news detection. Similarly, [33] compared linguistics and topical methodologies too discern between hyperpartisan and neutral news. That was one of the first work in literature and defined the importance of linguistics features in this task. 14 additional papers were included after exploring similar works and citations thanks to this procedure. Lastly, we compared the several definitions of hyperpartisan news to stress the importance of having a specific and clear task not overlapping with related ones. Our work offers an extensive and comprehensive investigation of state-of-the-art techniques considering both mixed approaches, machine and deep learning application. To ensure our systematic review is both homogeneous and robust in terms of comparability, we focused on the most commonly used performance metrics in NLP: accuracy and F1 score. By collecting and analyzing these standard metrics, we aim to maintain consistency across the studies and enhance the reliability of our comparative analysis. Lastly, we retrieved and analyzed 38 datasets, reporting the evaluation metrics, embeddings and features used by researchers. Finally, we present some descriptive results regarding the trend of the publications over time (Fig 2) and the selected sample that highlight the main publishers (Fig 3).

### Transparency and replicability

Emphasis was placed on transparency and replicability to adhere to rigorous academic standards and required by PLOS ONE’s policy on Data Availability. Thus, a GitHub repository stores the queries employed and described in the paper as well as the results of the screening process described above. This enables fellow researchers to replicate the methodology and verify the findings. The repository is accessible at https://github.com/MichJoM/Hyperpartisan_News_Detection_Systematic_Review/tree/main. In addition to the previous information, it contains the explanation of how missing data were handled.

## Hyperpartisan news detection: Description of the phenomenon

In this section, we begin by examining the definitions of hyperpartisan news detection found in the reviewed literature. We then delve into the various biases that are related to our investigated phenomenon and constitute it. Additionally, we examined the diverse hyperpartisan sources and we provide an overview of the application domains. Finally, we discuss the different strategies for labeling an entity as hyperpartisan.

### The problematics of the definition

#### Definition of hyperpartisanship.

The term *Hyperpartisanship* (https://claremontreviewofbooks.com/hyperpartisanship/) is not certified in any dictionary. A widely accepted definition considers hyperpartisan news as having an extreme bias toward a particular political ideology or party [18]. This type of news reporting often presents information in a highly sensationalized and one-sided manner, prioritizing ideological loyalty over objective reporting and critical analysis. This behavior denotes an extreme political allegiance to a party, leading to intense disagreement with the opposing faction [18].

#### Vagueness of the definition and overlap with similar tasks.

The minimalist definition of hyperpartisanship is widely adopted by computer scientists, who tend to simplify social phenomena models when applying automated detection [26]. Hyperpartisanship coexists within the broader category of junk news and shares characteristics with tasks such as political, ideological, and fake news detection [34]. Due to the vagueness of the definition, hyperpartisan headlines are often difficult to cluster within the misinformation set, and there is a lack of consensus on what precisely constitutes hyperpartisanship [35]. The perception of news as hyperpartisan can depend on the reader’s epistemic bubble [36]. Additionally, both left and right extremisms do not show significant stylistic differences, making hyperpartisanship a subject-shifting concept [33]. While humans can assess the degree of hyperpartisanship in a given text due to their cultural and linguistic awareness, machines lack this capability.

Hyperpartisan news detection often overlaps or is confused with other disinformation tasks, such as fake news detection [19,37–40,94], and stance detection [41]. Specifically, hyperpartisanship might be conveyed through elements of fake news, aimed at propagating a specific agenda and manipulating readers to adopt a particular position on a given topic [40].

#### Traits of hyperpartisan news.

From a linguistic perspective, hyperpartisan articles exhibit a high count of adjectives and adverbs [42,43], extensive use of pronouns, and words of disgust [44]. These articles tend to feature longer paragraphs written in a sensationalist style, full of emotional language and rare terms [45]. Right-wing media, in particular, often employ hyperpartisan headlines, corroborating earlier findings [46,47]. Hyperpartisan news articles display hyper-polarized linguistic traits in their titles as well. However, hyperpartisanship opposes to balanced news, which are intended to report facts with balanced tone and informative intention.

#### Analogue biases.

Hyperpartisan news detection is a task in which certain textual features indicated above suggest that the writer is expressing an extremist, one-sided opinion. Moreover, various degrees with which typologies of bias occur contribute to make the text hyperpartisan. There are several taxonomies proposals for junk news like [28,34]. We will use the bias categories collected by Oxford (https://catalogofbias.org/biases/spin-bias/) and [29] to discuss the founding biases of hyperpartisan articles.

*Spin bias*, or *rhetoric bias* [29], strictly concerns the linguistic structure of the article, its persuasion. The deliberate or inadvertent misrepresentation of research outcomes, leading to unjustified indications of positive or negative results, potentially could result in misleading conclusions. Written language is the product of the conscious application of strategic discursive and persuasive patterns to interest the readers. The words contribute to giving a particular meaning to the entire text, especially if they leverage an emotional lexicon with superlatives.

*Ad hominem bias* is a rhetorical strategy in which one moves away from the topic of the controversy by contesting not the statement of the interlocutor, but the interlocutor themselves and his personal characteristics or traits [48]. This rhetorical strategy was frequently used in sophistry and is still widely used today in political discussions and journalistic controversies.

*Presence bias* or opinion statement involves the inclusion of subjective opinions within news articles, influencing readers’ perceptions. It occurs when factual reporting is mingled with subjective viewpoints or opinions [49]. In other words, it reflects the degree of agreement and statement sharing of an entity, i.e. users or publishers [50].

*Ideological bias* occurs when news reporting or content is influenced by a particular ideological stance or viewpoint, impacting the presentation and selection of news topics. Ideological detection is different from political bias because some ideologies can be shared even by opposite parties. Ideologies often contrast each other, but to be classified they need this comparison [51].

*Framing bias* involves presenting information to shape or influence people’s perceptions of an issue or event by emphasizing certain aspects while downplaying others [52,120]. In this case, using linguistics and rhetorical figures helps the author partially present the selected information. Therefore, framing expresses a publishers leaning towards an ideology. Frames are tools that emphasize specific information while potentially favoring one aspect over another, with or without being slanted [53]. It is performed in moral content and style used [21].

*Coverage bias*, is not present in Table 2 since it is not a textual bias. It refers to the disproportionate attention or neglect of topics or events in news reporting, leading to an imbalance in coverage across different subjects [54].

**Table 2:** Examples of statements for specific biases and hyperpartisan statements for that bias.

Bias	Biased Example	Hyperpartisan Biased Example
Spin bias	How can we trust their solutions when they fail to understand the basic principles of economics?	Their economic policies are a disaster, proving yet again their ignorance and incompetence.
Ad hominem	The proposal is flawed because it comes from someone who has no experience in the field.	We can’t expect anything good from someone who has never done anything worthwhile!
Opinion statement bias	It’s clear that this is the best approach for our society.	This is unequivocally the only right path forward for our nation; anyone who disagrees is simply blind to the truth.
Ideology bias	Socialist policies always lead to inefficiency and economic downfall.	The leftist agenda destroys economies and personal freedoms every time it’s implemented.
Framing bias	The data support that this policy will decrease crime rates.	The indisputable facts confirm that only this policy can save us from spiralling into a crime-infested nightmare.
Political bias	This party’s proposal will bankrupt the nation.	The opposition’s plan is a surefire way to plunge our country into insurmountable debt and chaos.

*Political bias* could be easily confused with ideological bias. Since a party is a combination of both an ideology and a political leaning, this bias is related to the inclination of news media or information sources or people to favor one political party’s agenda [55].

In this context, it is essential to avoid conflating the reification of the social phenomenon involving linguistic indicators with the entirety of the specified biases. Namely, not all categories of biases mentioned can be classified as hyperpartisan when they manifest. The linguistic element of exaggeration *per se* does not automatically denote hyperpartisanship; rather, it necessitates contextual positioning, such as aligning with a particular party or ideology. Simply adopting a stance is insufficient for categorization as hyperpartisan; it is the degree of exaggeration in that stance that holds significance. We propose some examples to illustrate this in Table 2.

#### Proposal for a definition.

By reviewing the various definitions collected in Table 3, several key observations emerge:

- the concept of hyperpartisanship is an intersected field and shares features with the typologies of media biases discussed in section *Analogue bias*. In the following list the indexes define the intrinsic characteristics in the hyperpartisan definitions in 3, “Characteristic" column:
  1. spin bias;
  2. ad hominem bias;
  3. opinion statement bias;
  4. ideology bias;
  5. framing bias;
  6. coverage bias;
  7. political bias;
- it is commonly acknowledged that hyperpartisan news exhibits one-sided political bias, incorporating specific statements aligning with the ideology of a particular political party and/or agenda;
- the lack of a commonly shared definition across various studies results in the characteristics of detection being variable and mutually exclusive undermining the integrity and scientific rigor of research in this field;
- while approaching this classification task, some researches like [56] lack a methodological approach because do not introduce a definition of the phenomenon.

**Table 3:** Definitions of hyperpartisanship given in the selected papers.

Reference	Claim	Characteristics
[57]	Social media also have facilitated the rise of hyperpartisan news. Hyperpartisan news: (1) has an obviously one-sided political agenda, which makes no effort to balance opposing views; (2) pushes anti-system messages that are critical of both mainstream media and establishment politics, often relying on misinformation to do so; and (3) relies heavily on social media as a platform for dissemination. Thus, hyperpartisan news can be situated squarely at the intersection of partisan and alternative news, and considerable overlap exists between hyperpartisan news and “fake” news.	1, 6, 7
[18]	Hyperpartisan articles mimic the form of regular news articles but are one-sided in the sense that opposing views are either ignored or fiercely attacked.	1, 2, 3, 4, 5, 7, 8
[33]	Prone to misunderstanding and misuse, the term “fake news” arose from the observation that, in social media, a certain kind of “news” spreads much more successfully than others, and this kind of “news” is typically extremely one-sided (hyperpartisan), inflammatory, emotional, and often riddled with untruths.	1, 3, 4, 5, 7, 8
[46]	We think that a better understanding of hyperpartisanship can be achieved by considering not only (1) the news that contains one-sided opinions but also (2) the news that describes conflicts and the underlying politically polarized climate because both of them could lead to an increase in the public’s perceived polarization (Yang et al. 2016; Fiorina, Abrams, and Pope 2005; Levendusky and Malhotra 2016). Additionally, coverage quantity itself can be considered as a particular form of bias (Lin, Bagrow, and Lazer 2011). In particular, we seek to extend previous studies’ definitions of hyperpartisan news to include news that covers partisan conflicts and confrontations.	1, 3, 4, 7
[58]	Media bias can be observed and defined through various factors. In the political domain, it ranges from selectively publishing articles to specifically choosing to highlight some events, parties and leaders. We also come across articles where bias can be detected by observing the unclear assumptions, loaded language, or lack of proper context.	1, 6, 7, 8
[59]	Researchers use different terms to indicate the same issue, namely disinformation, misinformation, propaganda, junk news and click-bait. In this work we use the word disinformation, rather than the more popular “fake news”, to refer to a variety of low credibility content which comprises false news intended to harm, misleading and non-factual reporting, hyper-partisan news and propaganda, and unverified rumours	1, 4, 7
[60]	Hyperpartisan news is news riddled with untruth and twisted statements of information. This type of news spread more successfully than others. Hyperpartisan news not only can mislead readers but also cause polarisation within a community or society.	1, 4, 7
[61]	Although there are more subcategories related to fake news such as satire, parody or clickbait, a general definition of the term could be the following: fake news represents a way to spread false information to mislead the public, damage the reputation of an entity or have a political or financial gain. The idea of misleading and influencing the public is also linked to the notion of hyper-partisan news that has the role of presenting extremist or conspiratorial opinions with intentional misconceptions.	2, 3, 4, 5, 7
[37]	The aim is to classify news as real or fake. Fake news is news that is intentionally generated to misguide people. It may exist in various forms, including misleading content, biased news, satirical news, rumours, hyper-partisan news, deceptive news, disinformation, clickbait, and hoax.	1, 4, 7

In light of these considerations, hyperpartisan detection must necessarily consider different variables simultaneously: positioning, presence of a bias and its degree of exaggeration. Does the current state of the art in detection methodologies do this? As mentioned earlier, a detection method that simultaneously considers the different types of biases and these three variables has not been conducted. Various research works tend to focus individually on specific subsets of linguistic and content-based features, as outlined in the following sections.

Considering these elements, we propose the following definition to aid future research in addressing hyperpartisan news in Computer Science: Hyperpartisan news detection is the process of identifying news articles that exhibit extreme one-sidedness, characterized by a pronounced use of bias. The prefix "hyper-" highlights the exaggerated application of at least one specific type of bias—such as spin, ad hominem attacks, opinionated statements, ideological slants, framing, selective coverage, political leaning, or slant bias—to promote a particular ideological perspective. This strong ideological alignment is conveyed through amplified linguistic elements that reinforce one of these bias types within the text.

### Where can hyperpartisanship be detected? Perspectives on the sources

In this section, we will give a general overview of the main sources typologies considered to detect hyperpartisan news articles.

In light of the prevalence of hyperpartisan news dissemination online, the methodologies discovered are implemented specifically on online news outlets. Initially, when considering the domain of publishers, a linguistic approach can be applied to news analysis to detect hyperpartisanship. This approach involves studying textual information within articles using style-based or topic-based models [33,46,62,128]. Detection methods consider specific sections, such as the title [46,47], sentences [63], quotes in the body [42], or encompass both [46,58,64,65,94]. Otherwise, researchers investigated hyperpartisanship spread starting from entities involved in the writing and publishing process, like journalist’s [66] or media [18] leaning . Considering publishers as entities often interconnected through economic and political bonds [67], they form a polarized network, which can be analyzed using metadata like external links [68–70,138]. While determining bias based on the source is feasible [66,71], an article from a biased media outlet may not always be hyperpartisan [49,72]. This issue was underscored by [72], which highlighted the inadequacy of the information source in determining an article’s hyperpartisanship. This method generates a system capable of indicating bias scores in news and suggesting similar topics from different sources to encourage readership of diverse perspectives or to avoid extremely biased news.

Working with textual data enables the extraction of sentiment features [73]. For instance, [74] observed that sentiment analysis, applied to titles and sentences using TextBlob (https://textblob.readthedocs.io/en/dev/), improved evaluation metrics. Additionally, [75] noted that hyperpartisan articles tend to convey more aggressive and negative sentiments compared to other articles. Using VADER (https://github.com/cjhutto/vaderSentiment), [76] conducted experiments to analyze the contribution of sentiment features in indicating the author’s bias. Meanwhile, [77] approached hyperpartisan news detection by considering sentiment as a means to capture the polarity of articles.

Moreover, [78] employed both textual and image features to detect hyperpartisanship. Their study revealed that automated methods outperformed humans and that incorporating additional information such as images and titles enhanced the accuracy of the model.

### How hyperpartisanship is labeled?

Understanding the measurement of hyperpartisanship involves considering the diverse scales utilized. In Social Sciences, a range of indexes and scales is employed for this purpose, leveraging distinct features from those used in automatic detection methodologies. For instance, polarization is calculated with the CSES Polarization Index. The Common CSES Polarization Index (PI) is a tool used to assess the distribution of political parties across the Left/Right ideological spectrum. These metrics gauge ideological positioning and account for party sizes or vote shares, offering a comprehensive view of ideological stance and political influence [3]. Differently, automatic hyperpartisan detection relies on linguistic features. Some studies employ binary classification methods, utilizing labels such as hyperpartisan/mainstream (i.e. non-hyperpartisan) [18], Left/Right [66,118]. However, such distinctions often overlook nuanced differences within diverse political leanings [33]. Few studies have extended their scope to include a more fine-grained polarization range [79]. For example, [80] approached hyperpartisan detection as a multi-class classification problem, employing both 7- and 5-point scales to define affiliations: 1-2.5 – far-left, 2.5-3.5 – center-left, 3.5-4.5 – center, 4.5-5.5 – center-right, 5.5-7 – far-right. Similarly, [73] used a scale and [81] sought to manage granularity by distinguishing between right, center, and left affiliations.

## Approaches for automatic hyperpartisan news detection

The detection of hyperpartisan content encompasses a range of methodologies, varying from traditional non-deep learning approaches to cutting-edge deep learning techniques, as well as mixed learning algorithms. Non-deep learning methodologies often rely on traditional machine learning algorithms, leveraging handcrafted features and rule-based systems to identify linguistic patterns, stylistic markers, and network structures within textual and metadata sources. These approaches commonly include stylometric analysis and topic modeling methods to discern biased content. In contrast, deep learning methodologies harness the power of neural networks to automatically extract intricate features from raw data, enabling the identification of complex patterns and relationships in unstructured text or network data. These techniques, such as convolutional neural networks (CNNs), recurrent neural networks (RNNs), and Transformers, excel in learning representations directly from the data.

### Models discussion

In the following subsections, to encompass the risk of bias, we grouped and discussed the mentioned studies by model architecture. We will differentiate between Non-deep learning, Deep learning, and other methodologies adopted in the papers selected for the systematic review. The Deep Learning section includes Non-transformer’s Deep Learning models and the Transformers family. In the following tables: 4, 5, 6 and Table 7, we categorize the best model for each paper, reporting its performance. When researchers compared more models on the same task using different datasets, like the case of [118], we report only the best model’s performance.

#### Non-deep learning methods.

In Table 4, we categorized papers using the traditional machine learning approaches. The methodologies involved algorithms like Support Vector Machines, Random Forest, and Logistic Regression, followed by Linear regression, Naive Bayes, Linear SVC, KNN, XGBoost, and Maxent.

**Table 4:** This table describes the traditional Machine Learning algorithms used in the selected literature.

Algorithm	Reference
Support Vector Machine (SVM) [82]	[42–44,70,76,83,94]
XGBoost [84]	[85]
Maxent [86]	[40]
Random Forest [87]	[64,80,88–90]
Naive Bayes	[47,74]
Copula Ordinal Regression	[73]
Logistic Regression	[37,45,77,91,92]
Linear Support Vector Classifier (SVC)	[75]
Linear Classifier	[93]

There are effective strategies adopted with the SVM model. For instance, [70] used this algorithm combined with the sentiment analysis via National Council Canada’s Emotion Lexicon (NRC Emotion Lexicon) to analyze the emotional content in the article. Moreover, they extended the linguistic approach applyinh Linguistic Inquiry and Word Count (LIWC). They also considered the articles’ structure and meta-data as features. [94], adopted n-grams, i.e. bi- and tri-grams, and dependency sub-trees that impacted the performance. On the other hand, [83] experimented several embeddings: Doc2Vec [95], Glove [96], ELMo [97]. They found out that “adding simple lexical and sentiment features hurts the performance". [43] studied the linguistics divergencies between fake and hyperpartisan news employing an SVM. In this case, it emerged that hyperpartisan articles exhibit more sentences and a higher adjective count compared to unbiased news. When comparing the characteristics of extreme polarized articles against fake news, they noted that the former contains high usage of question/exclamation marks and adjectives. These sentence-related features delineate distinct linguistic patterns. [77] confirmed the robust potentialities of the Logistic Regression, ranking in the second place at the SemEval-2019. [77] built representations with Universal Sentence Encoder (USE) [98] and combined both semantic and handcrafted features, paying attention to the grade of the adjectives and subjectivity and distinguishing between two levels of polarity: sentence and article level. [37] used the Reuter Dataset for the training and the test, combining the ELMo embedding with a logistic regression classifier as already done by [72] and [77], confirming the effectiveness of this method. [77] discovered that the most relevant features concern bias lexicon and polarity. [93] placed third at the SemEval-2019 and found that article length was a distinctive trait of biased articles. By working at the phrase level, they created a set of phrases to discern the different types of articles, paying attention to removing n-grams containing publishers’ style biases. [46] focused on news titles with a topic-based approach. They also built a dataset considering two distinct typologies of news titles, augmenting the granularity of the detection. The first category pertains to descriptions of confrontations or conflicts between opposing parties, suggesting a deeply polarized political climate. The second set involves opinions that express a biased, inflammatory, and aggressive stance against a policy, a political party, or a politician. [73] thought there was an interdependence between factuality and political ideology bias, so that introduced a multi-task learning setup with the Copula Ordinal Regression (COR) [99]. They used the entire news outlet and considered diverse scales for measuring factuality (3-point scale) and political bias (7-point scale). [40] with Maximum Entropy Modeling (MaxEnt) by-passed linguistics features to build a model capable of generalizing as much as possible, [40] devised a document classification system that combines clustering features with simple local features. They showcased the effectiveness of employing distributional features from large in-domain unlabeled data. [85] approached the task using n-gram embeddings with article and title polarity, implementing the XGBoost model with all of these scalar features, but it performed poorly. They derived their methodology of applying stylometric analysis from [33]. This approach utilized n-grams, readability scores and Part-of-Speech (PoS) followed by binary classification. Thanks to unmasking information, they simultaneously compared documents with opposite political leaning. In doing so, [33] investigated the style variations depending on the political orientation and confronted it with a topic-based bag-of-words models. This methodology highlighted the limited usefulness of integrating corpus characteristics when performing a granular distinction amongst left, right and mainstream styles. Indeed, both the political extremes show similarities and can produce confounding effects in the model. Hence, concerning the style analysis for the hyperpartisan detection, the categories should be limited to mainstream and hyperpartisan without considering the specific leaning.

Furthermore, for a complete understanding of the approaches used in the literature, we summarized them in the Table 5. In this case, although ELMo, BERT, and Word2Vec embeddings were used as features of Non-Deep Learning algorithms. Table 5 describes only the features used gwith the best models proposed in Non-Deep Learning approaches in Table 4. We distinguish between features (Morpho-syntactic, Lexicon, Semantic, Sentiment and Metadata) and approaches (style-based and topic-based).

**Table 5:** Features used with the best models described in Table 4. The features described in the columns are the following: Morpho-syntactic (MS), Lexicon (L), Semantic (S), Sentiment (SE) and Metadata (M). The approaches are: Style-based (SB) and Topic-based (TB).

Reference	Method	Model	Dataset	Lang.	CM	Acc.	F1	MS	L	S	SE	M	SB	TB
Naredla, N.R., 2022	ML	RF	SE19	Eng	U.S.	.88	U/A			ELMo emb			x	
Garg, S., 2022	ML	LR	Reuter	Eng	U.S.	U/A	.93			ELMo emb			x	
Dumitru, V.C., 2019	ML	LSVM	Own	Eng	U.S.	.93	.81			TF-IDF		x	x	
Srivastava, V., 2019	ML	LR	SE19	Eng	U.S.	.82	.82	x	x	USE	x		x	
Hanawa, K., 2019	ML	LC	SE19	Eng	U.S.	.81	.80			BERT emb			x	
Yeh, C.L., 2019	ML	SVM	SE19	Eng	U.S.	.80	.79			BoW				
Palić, N., 2019	ML	SVC	SE19	Eng	U.S.	.79	.76	x	x	Word2Vec	x	x		
Stevanoski, B., 2019	ML	RF	SE19	Eng	U.S.	.77	.74	x	x	Word2Vec		x	x	
Nguyen, D.V., 2019	ML	SVM	SE19	Eng	U.S.	.75	.74	x		N-grams			x	
Chen, C., 2019	ML	NB	SE19	Eng	U.S.	.74	.74	x	x	BoW	x			
Agerri, R., 2019	ML	Maxent	SE19	Eng	U.S.	.74	.73	x		Word2Vec		x		
Alabdulkarim, A., 2019	ML	SVM	SE19	Eng	U.S.	.74	.71	x	x	TF-IDF	x	x	x	
Saleh, A., 2019	ML	LR	SE19	Eng	U.S.	.73	.73	x	x	BoW			x	
Bestgen, Y., 2019	ML	LR	SE19*	Eng	U.S.	.70	.68	x		BoW				
Knauth, J., 2019	ML	SVM	SE19	Eng	U.S.	.67	.69	x	x				x	x
Cruz, A., 2019	ML	RF	SE19	Eng	U.S.	.72	.67	x		BoW			x	
Sengupta, S., 2019	ML	LR	SE19	Eng	U.S.	.70	.68			unigrams				
Amason, E., 2019	ML	NB	SE19	Eng	U.S.	.65	.63	x	x	BoW	x		x	
Anthonio, T., 2019	ML	SVM	SE19	Eng	U.S.	.62	.69	x	x		x			
Chakravartula, N., 2019	ML	RF	SE19*	Eng	U.S.	.61	.66			BoW				
Gupta, V., 2019	ML	XGBoost	SE19	Eng	U.S.	0.55	0.28	x		N-grams	x		x	
Baly, R., 2019	ML	COR	MBFC	Eng	U.S.	U/A	U/A	x	x	Word2Vec				

#### Deep learning methods.

In the following paragraphs, we analyzed the Deep Learning methods adopted by diverse authors to solve the hyperpartisan detection task. In Table 6, we categorized papers using the traditional machine learning approaches. Lastly, at the end of the section, a comprehensive Table 7 collects and illustrates the results rounded to two decimals reported by all the authors studied in our systematic review.

**Table 6:** Collection of the most performant deep learning models used in the literature.

Algorithm	Description	Reference
Recurrent Neural Network-based (RNN) [100]	Recurrent Neural Networks are a class of artificial neural networks designed to process sequential data by maintaining an internal memory. They possess loops that allow information persistence, enabling them to consider past inputs for present computations, making them adept at handling time series or sequential data in various applications.	[51]
Mesh Neural Network [79]	It is a neural network aiming at maximazing the weight of past examples. Its structure is composed by recurrent nodes that boost the inductive abilities of the machine.	[79]
Catboost [101]	Catboost stands out as a specialized library crafted for boosting gradients. It employs a unique algorithm to prevent target leakage, ensuring high efficiency and precision.	[81]
Convolutional Neural Network	Convolutional Neural Networks were designed for image processing but were applied to Natural Language Processing, in which the input text is a one-dimensional vector of tokens, or like a matrix of embeddings. This model can learn both local and global dependencies as well as hierarchical features in the data.	[42,65,72,102–105,138]
Hierarchical Attention Network (HAN) [106]	The HAN is a model capable of balancing the information in a current state, deciding whether to update it and how much the past information contributes to its new state.	[58,66,107–109]
Long Short Term Memory-based models [110]	Unidirectional LSTM retain information solely from preceding inputs as it has only processed data from the past. Employing a bidirectional approach involves processing inputs in two directions: from past to future and from future to past. The distinctive aspect of this method, compared to unidirectional LSTM, lies in the LSTM running in reverse, which captures information from the future. By amalgamating the two hidden states, this technique enables the retention of information from both the past and future at any given time. In NLP, the main difference lies in the context captured. Bi-LSTM can discover hidden reversed semantic and syntactic structures.	[111–114,120]
RoBERTa [115]	RoBERTa, an extension of BERT, refines language understanding by optimizing training techniques, removing sentence order prediction, and leveraging large-scale data. It enhances pre-training for improved performance across various language tasks, achieving state-of-the-art results in natural language processing.	[116–118,128]
BERT [119]	BERT (Bidirectional Encoder Representations from Transformers) is a pre-trained language model by Google, adept at understanding the context in both directions of a sentence. It utilizes Transformer architecture, enabling versatile language understanding for diverse tasks like question answering and natural language understanding.	[46,49,60,62,105,118,121–127,129,132,137,139]

**Table 7:** This table describes the best performances of the models.

Reference	Dataset	Language	Media’s Country	Method	Model	Accuracy	F1
[46]	Own	English	U.S.	DL	BERT	0.84	0.78
[129]	Own	Persian	Iran	Other	ChatGPT	0.85	0.85
[109]	Semeval-2019	English	U.S.	DL	HAN	0.95	U/A
[116]	Semeval-2019	English	U.S.	DL	MP-tuning	0.91	U/A
[128]	Semeval-2019	English	U.S.	DL	RoBERTa	0.84	0.83
[124]	Semeval-2019	English	U.S.	DL	BERT	0.83	U/A
[125]	Semeval-2019 by-article + by-publisher	English	U.S.	DL	BERT	U/A	0.91
[81]	Task 3A	English	U.S.	DL	CatBoost	0.69	0.69
[64]	Semeval-2019	English	U.S.	ML	Random Forest	0.88	U/A
[117]	Semeval-2019	English	U.S.	DL	RoBERTa	0.85	0.85
[118]	Framing Triplet Dataset	English	U.S.	DL	multi-task BERT-based	0.84	U/A
[79]	Own	English	U.S.	DL	Mesh Neural Network	0.45	U/A
[66]	Presidential	English	U.S.	DL	HAN	0.91	0.90
[37]	Reuter	English	U.S.	ML	Logistic Regression	U/A	0.93
[132]	Stereoimmigrants	Spanish	U.S.	DL	BERT	0.86	0.83
[62]	BuzzFeed-Webis Fake News	English	U.S.	DL	BERT	0.89	0.86
[112]	Semeval-2019	English	U.S.	DL	LSTM	0.86	0.84
[102]	BuzzFeed-Webis Fake News	English	U.S.	DL	CNN	U/A	0.73
[105]	Semeval-2019 by-article + by-publisher	English	U.S.	DL	BERT	0.87	0.81
[80]	GERMAN Dataset	German	Germany	ML	Random Forest	U/A	0.79
[107]	Semeval-2019	English	U.S.	DL	HAN	0.82	0.81
[120]	Own	English	U.S.	DL	LSTM	U/A	0.80
[49]	PoliNews	English	U.S.	DL	BERT	U/A	U/A
[51]	Own	English	India	DL	RNN	0.84	0.87
[121]	Own	English	U.S.	DL	BERT	0.72	U/A
[43]	Own	English	U.S.	ML	LSVM	0.93	0.81
[72]	Semeval-2019	English	U.S.	DL	CNN	0.82	0.81
[77]	Semeval-2019	English	U.S.	ML	Logistic Regression	0.82	0.82
[93]	Semeval-2019	English	U.S.	ML	Linear Classifier	0.81	0.80
[111]	Semeval-2019	English	U.S.	DL	LSTM	0.80	0.80
[83]	Semeval-2019	English	U.S.	ML	SVM	0.80	0.79
[75]	Semeval-2019	English	U.S.	ML	SVC	0.79	0.76
[122]	Semeval-2019	English	U.S.	DL	BERT	0.78	0.77
[127]	Semeval-2019	English	U.S.	DL	BERT	0.78	0.76
[126]	Semeval-2019	English	U.S.	DL	BERT	0.77	0.75
[90]	Semeval-2019	English	U.S.	ML	Random Forest	0.77	0.74
[137]	Semeval-2019	English	U.S.	DL	BERT	0.76	0.76
[94]	Semeval-2019	English	U.S.	ML	SVM	0.75	0.74
[40]	Semeval-2019	English	U.S.	ML	Maxent	0.74	0.73
[74]	Semeval-2019	English	U.S.	ML	Naive Bayes	0.74	0.74
[138]	Semeval-2019	English	U.S.	DL	CNN	0.74	0.70
[42]	Semeval-2019	English	U.S.	DL	CNN + LSTM	0.74	0.71
[91]	Semeval-2019	English	U.S.	ML	Logistic Regression	0.73	0.73
[89]	Semeval-2019	English	U.S.	ML	Random Forest	0.72	0.67
[108]	Semeval-2019	English	U.S.	DL	HAN	0.72	0.69
[45]	Semeval-2019	English	U.S.	ML	Logistic Regression	0.70	0.68
[114]	Semeval-2019	English	U.S.	DL	LSTM	0.68	0.63
[104]	Semeval-2019	English	U.S.	DL	CNN	0.67	0.74
[47]	Semeval-2019	English	U.S.	ML	Naive Bayes	0.65	0.73
[123]	Semeval-2019	English	U.S.	DL	BERT	0.64	0.64
[76]	Semeval-2019	English	U.S.	ML	SVM	0.62	0.69
[103]	Semeval-2019	English	U.S.	DL	CNN	0.60	0.70
[113]	Semeval-2019	English	U.S.	DL	LSTM	0.58	0.68
[131]	Semeval-2019	English	U.S.	Other	Gagavai Explorer	0.56	0.68
[73]	MBFC	English	U.S.	ML	Copula Ordinal Regression	U/A	U/A
[85]	Semeval-2019	English	U.S.	ML	XGBoost	0.55	0.28
[139]	Semeval-2019	English	U.S.	DL	BERT	0.50	0.61
[92]	Semeval-2019 by-publisher	English	U.S.	ML	Logistic Regression	0.70	0.68
[65]	Semeval-2019 by-publisher	English	U.S.	DL	CNN	0.66	0.70
[88]	Semeval-2019 by-publisher	English	U.S.	ML	Random Forest	0.61	0.66
[60]	Semeval-2019 by-article + by-publisher	English	U.S.	DL	BERT	0.68	U/A
[58]	Telugu	Telugu	India	DL	HAN	0.89	U/A
[70]	Semeval-2019	English	U.S.	ML	SVM	0.74	0.71
[44]	Semeval-2019	English	U.S.	ML	SVM	0.67	0.69
[33]	BuzzFeed-Webis Fake News	English	U.S.	ML	U/A	0.75	0.78
[69]	Own	English	U.S.	Other	MVDAM	0.80	0.79

#### Deep learning: Non transformer-based architectures.

[42] employed a fusion of CNN and LSTM, utilizing quantitative linguistic features extracted through GloVe. In this way, they highlighted the crucial role of incorporating linguistic features alongside representations based on word vectors. Additionally, they built a meta-classifier to filter noisy data to apply to the by-publisher dataset. [72] won the SemEval-2019 Task 4 by combining rich morphological and contextual representations by averaging the three vectors per word into ELMo embeddings. Their model was used for further studies by [105] for pseudo-labeling frameworks: Overlap-checking and Meta-learning. Overlap-checking consists of adding data, helping the model train, while Meta-learning allows the model to be continually trained on a clean dataset and a pseudo dataset. This last work inspired [107]. In their article, they used a HAN combined with ELMo embeddings. The HAN is a model capable of balancing the information in a current state, deciding whether to update it and how much the past information contributes to its new state. In this case, the information stems from sentence level, confirming that richer article representations yield better performances. By encapsulating the articles’ structure, connectors and paying attention to stylistic markers, handcrafted stylistic features and emotion lexicons, they reached the state-of-the-art in 2020 on the SemEval-2019 Task 4 dataset. [109] improved the HAN standard model by introducing Knowledge Encoding (KE) components. The HAN segment functions to grasp word and sentence relationships within a news article, employing a structured hierarchy across three levels—word, sentence, and title. Meanwhile, the KE component integrates common and political knowledge associated with real-world entities into the prediction process for determining the political stance of the news article. Since the model is not language-based, it could work with diverse languages beyond English. [112] developed a pre-training framework encoding knowledge about entity mentions, namely masked tokens as frame indicators, and modeling the propagation between users with a social information graph. They noted that models pre-trained on general sources and tasks have limited ability to focus on biased text segments. [113] introduced a voting system of LSTMs to build a controlled dataset to train another LSTM. It was an example to demonstrate the importance of having a balanced and clean dataset to run experiments. Lastly, [120] built a Hierarchical-LSTM applied to subframes (n-grams) to tackle the framing bias. In this paper, they introduced a pioneering framework aimed at pretraining text models utilizing signals derived from the abundant social and linguistic context available, encompassing elements such as entity mentions, news dissemination, and frame indicators.

#### Deep learning: Transformer-based architectures.

Regarding the Transformers architectures, we observed a massive utilization of BERTbase and BERTlarge. BERTbase is a pre-trained BERT model trained on a smaller dataset than BERTlarge. BERTbase differentiates itself in cased and uncased, depending on whether to discern between cases and uncased words. [121] wanted to remove the bias when modeling the medium. They observed that combining bias mitigation with triplet loss, Twitter bios and media-level representations increased the model efficacy. [118] proposed a multi-task BERT-based model with contrastive learning to tackle framing bias in news articles. [122] with BERT and combinations of syntactic bigram counts and psycholinguistic features investigated the inference of political information and hyperpartisanship on author and text level starting from linguistic data. [123] showed that fine-tuning the model entails better results. [124] introduced a semi-supervised framework trained using federating learning, namely algorithms are trained independently across diverse datasets. Furthermore, textual data are tagged to extrapolate wh-questions replies and temporal lexicon information. The same author replicated this approach in [125]. In the quest for precise detection and data denoising, the same author replicated this approach with variations in [125]. [125] employed an attention-based strategy to learn text representation, aiming to identify target expressions accurately while extracting pertinent contextual information. They generated a BERT attention embedding query utilizing lexicon expansion, content segmentation and temporal event analysis. Ultimately, this approach enhances the understanding of consecutive news articles within a temporal framework. [126] experimented using BERTbase and BERTlarge feeding them with embeddings of different lengths. They were interested in analyzing the parts of the articles, looking for a consistent level of hyperpartisanship that demonstrated to exist. [60] from the confrontation between BERT and ELMo models, confirmed that the inputs and embeddings dimensions contributed to affecting positively the performance. [127] performed domain adaptation, showing its efficacy. [116] operated in a low-resource scenario with prompt-based learning and employed masked political phrase prediction and a frozen pre-trained language model that relies on transformer architecture, utilizing the robustly optimized BERT approach known as RoBERTa as a backbone for their own model, MP-tuning. [117] focuses on political ideology and stance detection, comparing triplets of documents on the same history to detect dissimilarities amongst them. They trained RoBERTa through continual learning. Whereas, [128] improved their model’s performance with cross-domain contrastive learning and this work is noticeable that they used GPT-2 for augmenting hyperpartisan textual data. Lastly, [129] faced the task for Persian hyperpartisan tweets by prompting GPT-3.5, a multi-language conversational generative LLM released in 2022, and open-weights model like Llama2 [130]. [129] compared the capabilities of Large Language Models (LLMs) and BERT-based models like RoBERTa and ParseBERT to detect English and Persian tweets, providing instructions with different levels of specifity to the models. Despite the huge dimension and the extensive training of LLMs, fine-tuning ParseBERT and RoBERTa has proven to be more efficient and practical for certain tasks.

#### Other methods.

Within the vast landscape of computational frameworks, certain algorithms defy classification within the traditional realms of deep learning or non-deep learning. This chapter delves into the exploration of these unique frameworks—sophisticated combinations of diverse models, labeling techniques and graph approaches—that operate beyond the conventional boundaries of established categorizations.

[49] applies a framework for presentation bias, studying hyperpartisanship with a graph-based method. This three-step framework is so structured: collecting related-articles clusters on the same topic; applying Aspect-based Sentiment Analysis (ABSA) with BERTbase to rate and classify fine-grained opinions in the pairs of sentences; the variation in bias between news sources within similar categories is figured out by contrasting the scores of matching pairs of articles. This comparison is done for every combination of news sources within these categories, and the differences in bias are averaged across all article groups. This averaging process leads to the development of a bias matrix. [69] proposed a Multi-View Document Attention Model (MVDAM) capable of modeling at the same time title, structure and metadata like links in order to estimate the political ideology of a news article. This framework based on the Bayesian approach utilizes different models for creating the 3-D representation: a convolutional neural network for learning the title, Node2Vec for the network and HAN for the content. [131] worked mostly on manual features like metatopic, namely polarizing topics, using an end-to-end tool: The Gavagai Explorer, which performed poorly.

[33] performed a political orientation prediction and hyperpartisan classification task using an unmasking technique with binary classifiers. For the first task, they found that left-wing news tends to be easily misclassified. This study noticed that individual political orientation is struggling to predict and that a style-based approach overcomes the content-based one. Moreover, they discovered subtle differences in style between hyperpartisan news belonging to different political leanings. [62] using masking and transformer-based models proved that topic-based approaches lead to better results than style-based. Instead, [132] made a comparative examination of BERT-based models and masking-based models, enriching comprehension regarding the strengths and constraints of varied approaches in bias detection, offering crucial insights for upcoming research and advancements in this domain. In essence, these models’ contribution lies in their capacity to augment the precision, clarity, and comprehensibility of bias detection within political and social discussions. Consequently, they propel advancements in this pivotal research domain. Furthermore, [133] investigates using large language models for automated stance detection in a lower-resource language, focusing on immigration. It annotates pro- and anti-immigration examples to compare performance across models. The study finds that GPT-3.5 matches supervised models’ accuracy, offering a simpler alternative for hyperpartisan detection in media monitoring. Lastly, for the sake of exhaustiveness, we will briefly cover other methods not focusing on news textual features. For this reason, the following discussed papers are not included in our final selection. However, in this way, the reader can understand the complexities of approaches to tackle hyperpartisanship. [134] maps linguistic divergence across the U.S. political spectrum using 1.5M social media posts (20M words) from 10k Twitter users. By analyzing followers of 72 news accounts, it identifies variations in topics, sentiment, and lexical semantics. Methods combine data mining, lexicostatistics, machine learning, large language models, and human annotation. [135] analyzes language differences on Twitter among 5,373 Democratic and 5,386 Republican followers to explore psychological traits tied to political leanings. Using naturalistic data, it confirms hypotheses: liberals’ language shows uniqueness, swearing, anxiety, and emotions, while conservatives’ language reflects group identity, achievement, and religion, supporting prior research. To conclude, [136] introduced FAULTANA (FAULT-line Alignment Network Analysis), a computational method to identify societal fault lines and polarization drivers in online interactions. Using data from Birdwatch (Twitter) and DerStandard forums, it reveals two polarized groups aligned with political identities. FAULTANA tracks polarization over time, highlighting divisive issues and their impact. We present the best performances retrieved in the selected papers in Table 7.

## Datasets

In the previous section, we provided an overview of methodologies employed in addressing hyperpartisan detection. Effective models depend on top-notch data quality to function optimally. However, constructing a high-quality, well-balanced dataset can be both time-consuming and resource-intensive. This challenge is compounded by shifts in data policies across social networks since the Cambridge Analytica scandal, leading to potential difficulties or cost changes in obtaining data. Additionally, a trend has emerged within news sources where access to data is restricted due to its previous utilization in training models like GPT (https://www.washingtonpost.com/technology/interactive/2023/ai-chatbot-learning/). Consequently, news sources have implemented paywalls and crawler restrictions (https://ilmanifesto.it/termini-e-condizioni), making it exceedingly challenging to gather suitable information for this and similar tasks.

### Datasets presentation

To support upcoming studies on identifying hyperpartisan news and related tasks, we have created an extensive table: Table 8, which outlines key attributes of datasets relevant to hyperpartisan news detection. This table includes datasets referenced in different papers. Some are not primarily used for hyperpartisan detection but could be. It is important to note that when subsets or extended versions of earlier datasets exist, we consider them separate entities denoted by *. Additionally, datasets marked with ** signify merged collections. The column labeled *Data* indicates the number of articles gathered by the researchers.

**Table 8:** This table describes the datasets found in the literature.

Dataset	Reference	Year	Size	Bias Label	Language	Media’s country	Availability
No Name	[133]	2024	22628	U/A	Estonian	Estonia	No
No Name	[129]	2024	U/A	U/A	Persian	Iran	No
Task 3A	[81]	2023	55000	AllSides	English	U.S.	Yes
Task 3B	[81]	2023	8000	AllSides	English	U.S.	Yes
Allsides-L	[109]	2023	719256	Allsides	English	U.S.	Yes
No Name	[46]	2023	1824824	AllSides, Media Bias Factcheck	English	U.S.	No
Framing Triplet Dataset	[118]	2022	25627	Media Bias Factcheck	English	U.S.	Yes
No Name	[79]	2022	10000	Allsides	English	U.S.	No
TVP Info	[146]	2022	81694	U/A	Polish	Poland	Upon request
TVN 24	[146]	2022	128527	U/A	Polish	Poland	Upon request
BIGNEWS	[117]	2022	3689229	Allsides, adfontesmedia	English	U.S.	Upon request
*BIGNEWS BLN	[117]	2022	2331552	Allsides, adfontesmedia	English	U.S.	Upon request
*BIGNEWS ALIGN	[117]	2022	1060512	Allsides, adfontesmedia	English	U.S.	Upon request
GERMAN dataset	[80]	2021	47362	None	German	Germany	Yes
No Name*	[62]	2021	1555	BuzzFeed	English	U.S.	No
Stereo Immigrants	[132]	2021	3704	Manually labeled	Spanish	Spain	Yes
No Name*	[120]	2020	21645	Media Bias Factcheck	English	U.S.	Yes
The Annotated Data Dataset	[147]	2020	46	Media Bias Factcheck	English	U.S.	No
No Name	[59]	2020	37000	None	Italian	Italy	No
*PoliNews	[49]	2020	83000	U/A	English	U.S.	No
Presidential	[71]	2020	178572	Allsides, Media Bias Factcheck	English	U.S.	No
POLUSA	[141]	2020	9000000	None	English	U.S.	Yes
*Politifact	[61]	2020	18027	Politifact.com	English	U.S.	No
No Name	[51]	2020	4627	Manually labeled	English	India	No
No Name	[121]	2020	34737	Allsides	English	U.S.	Yes
All-Sides	[148]	2019	10385	None	English	U.S.	No
Telugu	[58]	2019	1327	Manually labeled	Telugu	India	Yes
NELA-2018	[142]	2019	713534	Allsides, Media Bias Factcheck, BuzzFeed et al.	English	U.S.	Yes
by-article	[18]	2019	1273	Manually labeled	English	U.S.	Yes
by-publisher	[18]	2019	754000	BuzzFeed news, Media Bias Factcheck	English	U.S.	Yes
BASIL	[143]	2019	300	Manually labeled	English	U.S.	Yes
The BuzzFeed-Webis Fake News Corpus 2016	[33]	2018	1627	BuzzFeed	English	U.S.	Yes
Reuter	U/A	U/A	18519	U/A	English	U.S.	Yes
No Name	[63]	2018	88	Crowd-sourcing	English	U.S.	No
MBCF	[144]	2018	1066	Media Bias Factcheck	English	U.S.	Yes
NELA-2017	[145]	2018	136000	U/A	English	U.S.	Yes
BuzzFeed 2016	buzzfeednews.com	2016	2282	BuzzFeed	English	U.S.	Yes
No Name	[52]	2015	74	Crowd-sourcing	English	U.S.	No

To provide comprehensive insights into the table, we will give a philological explanation of the datasets marked with the symbols * and **. *Framing Triplet Dataset* is a combination of the following datasets: SemEval-2019 task 4 along with [120]’s data. Furthermore, [120] expands the *SemEval-2019 task 4* dataset by incorporating articles collected from polarized sources and then labeled through mediabiasfactcheck.com. Regarding BIGNEWS, collected by [117], it has two subsets, respectively: *BIGNEWSBLN* is a downsampled corpus maintaining an equal distribution of ideologies, and *BIGNEWSALIGN*, which clusters news stories from opposing sources but on the same topic. In their research, [49] utilized a subset of *All-the-news* (https://www.kaggle.com/datasets/snapcrack/all-the-news). Furthermore, [33] worked with a subset of articles crawled from the URLs contained in *The BuzzFeed-Webis Fake News Corpus* collected by [140]. By cleaning [33]’s dataset, [62] obtained a new dataset. The same researchers created *StereoImmigrants*, a collection of Spanish news about immigrants, for [132].

Labeling and retrieving processes relied upon platforms like Allside (https://allsides.com/), Factcheck (https://mediabiasfactcheck.com/), Politifact (https://www.politifact.com/), as ground truth for establishing the bias of an article and as source where to collect data. Indeed, in these contexts, experts assign news to the political orientation.AllSides.com is a media company that specializes in providing balanced news coverage by collecting and comparing news stories from various sources with different political leanings. The platform categorizes news articles based on their political bias—whether left, center, or right—and scores them according to the level of partisanship they contain.

Since [121] noted that training models with big datasets reduce the performance due to their noise, researchers started to prefer the quality rather than the dimension. Indeed, [46], after a deeper analysis of the SemEval 2019 dataset, revealed several issues with this ground truth dataset widely used: class imbalance, task-label unalignment, and distribution shift.

**Fig 4:**
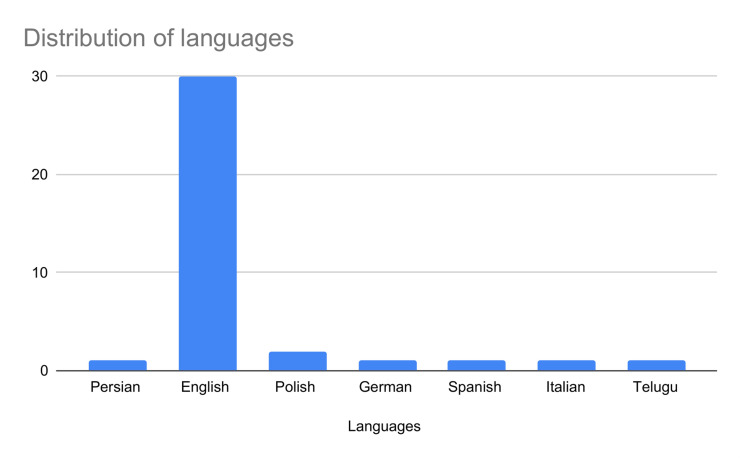
Language distribution in the datasets described in Table 8.

As we can see from Fig 4, there is an imbalanced distribution towards English data, leaving the context of minority languages understudied. Datasets are available at the respective links: [81] https://gitlab.com/checkthat_lab/clef2023-checkthat-lab/-/tree/main/task3?ref_type=heads, [109] https://github.com/yy-ko/khan-www23., [118] https://github.com/MSU-NLP-CSS/CLoSE_framing, [80] https://github.com/axenov/politik-news, [132] https://github.com/jjsjunquera/StereoImmigrants, [120] https://github.com/ShamikRoy/Subframe-Prediction, [141] https://urlis.net/zon9n8wr, [58] https://drive.google.com/drive/folders/1IyaKYeDkl7ubuabTI65G0nSBfxQNdeTr [142] https://dataverse.harvard.edu/dataset.xhtml?persistentId=doi:10.7910/DVN/ULHLCB, [18] https://zenodo.org/records/1489920 [143] www.ccs.neu.edu/home/luwang/data.html, [33] https://github.com/BuzzFeedNews/2016-10-facebook-fact-check, Reuter http://about.reuters.com/ researchandstandards/corpus/, [144] https://github.com/RWalecki/copula_ordinal_regression, [145] https://dataverse.harvard.edu/dataset.xhtml?persistentId=doi:10.7910/DVN/ZCXSKG, BuzzFeed https://github.com/BuzzFeedNews/2016-10-facebook-fact-check/blob/master/data/facebook-fact-check.csv.

### Potential limitations

The studies collected hold significant value, yet several inherent limitations in the datasets could influence the comprehensiveness and applicability of future findings. Firstly, the absence of a distinct dataset designed to differentiate between hyperpartisan and partisan news poses a fundamental challenge, potentially impacting the classification accuracy. Secondly, 2 shows that the predominant focus is on English news articles within the dataset. This fact raises concerns about minority languages and their respective democratic contexts, possibly skewing the representation and applicability of Anglo-American papers’ conclusions to their different socio-cultural environment. This discrepancy might lead to situations where certain democracies lack the necessary tools and datasets in their native language, hindering their ability to develop similarly effective analytical tools as over-represented democracies. Additionally, the phenomenon of hyperpartisanship varies significantly between countries due to the variety of party systems and different cultural backgrounds [6]. Consequently, the development of models trained on linguistically non-representative data may compromise their ability to efficiently detect hyperpartisanship in under-represented democracies, thereby impacting their success rates. Furthermore, issues pertaining to dataset maintenance, such as broken URLs, may impede replicability and accessibility for future research endeavors [33]. Furthermore, temporal lexicon constraints might hinder capturing shifts in textual patterns, tones, and context, affecting the accuracy of temporal analysis [46]. We highlight that cross-lingual comparison of hyperpartisan traits has never been studied from a computational approach. Thus, it is not possible to define if the online environment flattens cultural-linguistic traits pertinent to hyperpartisanship independently from the country and its political system. Another consideration regards the limited availability of data over time due to paywalls and copyright restrictions poses a significant barrier, potentially restricting the depth and breadth of future analysis within certain timeframes. Lastly, despite the popularity and the good results that researchers achieved, as far as we know, autoregressive models were not used.

## Conclusions and future works

In synthesizing insights from 81 studies, our systematic review illuminates the value of existing research in understanding hyperpartisan news. We summarized all the papers included in the systematic review in Table 9. With the support of this table, we are going to reply to the initial research questions.

**Table 9:** Table summarizing the papers selected with PRISMA methodology.

Reference	Year	Title	First Author	Dataset	Language	Media’s Country	Method	Model	Acc.	F1
[46]	2024	Computational assessment of hyperpartisanship in news titles	Lyu, H.	Own	English	U.S.	DL	BERT	0.84	0.78
[129]	2024	An evaluation of language models for hyperpartisan ideology detection in Persian Twitter	Omidi, S.	Own	Persian	Iran	Other	ChatGPT	0.85	0.85
[133]	2024	Automated stance detection in complex topics and small languages: The challenging case of immigration in polarizing news media	Mets, Mark	Own	Estonian	Estonia	Other	U/A	U/A	U/A
[109]	2023	KHAN: Knowledge-aware hierarchical attention networks for accurate political stance prediction	Ko	Semeval-2019	English	U.S.	DL	HAN	0.95	U/A
[116]	2023	Multi-stage prompt tuning for political perspective detection in low-resource settings	Kim, K.M.	Semeval-2019	English	U.S.	DL	MP-tuning	0.91	U/A
[128]	2023	From Fake to Hyperpartisan News Detection Using Domain Adaptation	Smădu, R.A.	Semeval-2019	English	U.S.	DL	RoBERTa	0.84	0.83
[124]	2023	Temporal positional lexicon expansion for federated learning based on hyperpatism detection	Ahmed, U.	Semeval-2019	English	U.S.	DL	BERT	0.83	U/A
[125]	2023	Semisupervised federated learning for temporal news hyperpatism detection	Ahmed, U.	Semeval-2019 by-article + by-publisher	English	U.S.	DL	BERT	U/A	0.91
[81]	2023	Frank at checkthat! 2023: Detecting the political bias of news articles and news media	Azizov, D.	Task 3A	English	U.S.	DL	CatBoost	0.69	0.69
[118]	2022	CLoSE: Contrastive learning of subframe embeddings for political bias classification of news media	Kim, M.Y.	Framing Triplet Dataset	English	U.S.	DL	multi-task BERT-based model	0.84	U/A
[64]	2022	Detection of hyperpartisan news articles using natural language processing technique	Naredla, N.R.	Semeval-2019	English	U.S.	ML	Random Forest	0.88	U/A
[117]	2022	POLITICS: pretraining with same-story article comparison for ideology prediction and stance detection	Lyu, Y.	Semeval-2019	English	U.S.	DL	RoBERTa	0.85	0.85
[79]	2022	An automated news bias classifier using caenorhabditis elegans inspired recursive feedback network architecture	Sridharan, A.	Own	English	U.S.	DL	Mesh Neural Network	0.45	U/A
[66]	2022	Political ideology detection of news articles using deep neural networks	M.Alzhrani, K.	Presidential	English	U.S.	DL	HAN	0.91	0.90
[37]	2022	Role of ELMo embedding in detecting fake news on social media	Garg, S.	Reuter	English	U.S.	ML	Logistic Regression	U/A	0.93
[146]	2022	Creation of Polish Online News Corpus for Political Polarization Studies	Szwoch, J.	TVP Info, TVP 24	Polish	Poland			U/A	U/A
[132]	2021	On the detection of political and social bias	Sónchez-Junquera, J.	Stereoimmigrants	Spanish	U.S.	DL	BERT	0.86	0.83
[62]	2021	Masking and transformer-based models for hyperpartisanship detection in news	Sónchez-Junquera, J.	The BuzzFeed-Webis Fake News Corpus 2016	English	U.S.	DL	BERT	0.89	0.86
[112]	2021	Using social and linguistic information to adapt pretrained representations for political perspective identification	Li, C.	Semeval-2019	English	U.S.	DL	LSTM	0.86	0.84
[102]	2021	Hyperpartisan news classification with ELMo and bias feature	Gerald Ki Wei, H.	The BuzzFeed-Webis Fake News Corpus 2016	English	U.S.	DL	CNN	U/A	0.73
[105]	2021	Bias bubbles: Using semi-supervised learning to measure how many biased news articles are around us.	Ruan, Q.	Semeval-2019 by-article + by-publisher	English	U.S.	DL	BERT	0.87	0.81
[80]	2021	Fine-grained classification of political bias in german news: A data set and initial experiments	Aksenov, D.	GERMAN Dataset	German	Germany	ML	Random Forest	U/A	0.79
[61]	2021	Topic-based Models with Fact Checking for Fake News Identification. - RoCHI - RoCHI	Dumitru, Vlad Cristian	Politifact	English	U.S.			U/A	U/A
[107]	2020	On document representations for detection of biased news articles	Cruz, A.F.	Semeval-2019	English	U.S.	DL	HAN	0.82	0.81
[120]	2020	Weakly supervised learning of nuanced frames for analyzing polarization in news media	Roy, S.	Own	English	U.S.	DL	LSTM	U/A	0.80
[49]	2020	How biased are american media outlets? a framework for presentation bias regression	Tran, M.	PoliNews	English	U.S.	DL	BERT	U/A	U/A
[51]	2020	Ideology detection in the indian mass media	Sharma, A.	Own	English	India	DL	RNN	0.84	0.87
[121]	2020	We can detect your bias: Predicting the political ideology of news articles	Baly, R.	Own	English	U.S.	DL	BERT	0.72	U/A
[43]	2020	Fake and hyper-partisan news identification	Dumitru, V.C.	Own	English	U.S.	ML	LSVM	0.93	0.81
[71]	2020	Ideology Detection of Personalized Political News Coverage: A New Dataset	Alzhrani, Khudran	Presidential	English	U.S.	U/A	U/A	U/A	U/A
[141]	2020	The POLUSA Dataset: 0.9M Political News Articles Balanced by Time and Outlet Popularity	Gebhard, Lukas	POLUSA	English	U.S.	U/A	U/A	U/A	U/A
[147]	2020	Creating a dataset for fine-grained bias detection in news articles	Lim, Sora	The Annotated Data Dataset	English	U.S.	U/A	U/A	U/A	U/A
[59]	2020	HoaxItaly: a collection of Italian disinformation and fact-checking stories shared on Twitter in 2019	Pierri, Francesco	Own	Italian	Italy	U/A	U/A	U/A	U/A
[72]	2019	Team Bertha von Suttner at SemEval-2019 task 4: Hyperpartisan news detection using ELMo sentence representation convolutional network	Jiang, Y.	Semeval-2019	English	U.S.	DL	CNN	0.82	0.81
[77]	2019	Vernon-fenwick at SemEval-2019 task 4: Hyperpartisan news detection using lexical and semantic features	Srivastava, V.	Semeval-2019	English	U.S.	ML	Logistic Regression	0.82	0.82
[93]	2019	The sally smedley hyperpartisan news detector at SemEval-2019 task 4	Hanawa, K.	Semeval-2019	English	U.S.	ML	Linear Classifier	0.81	0.80
[111]	2019	Dick-preston and morbo at SemEval-2019 task 4: Transfer learning for hyperpartisan news detection	Isibster, T.	Semeval-2019	English	U.S.	DL	LSTM	0.80	0.80
[83]	2019	Tom jumbo-grumbo at SemEval-2019 task 4: Hyperpartisan news detection with GloVe vectors and SVM	Yeh, C.L.	Semeval-2019	English	U.S.	ML	SVM	0.80	0.79
[75]	2019	TakeLab at SemEval-2019 task 4: Hyperpartisan news detection	Palić, N.	Semeval-2019	English	U.S.	ML	SVC	0.79	0.76
[122]	2019	Politically-oriented information inference from text	Da Silva, S.C.	Semeval-2019	English	U.S.	DL	BERT	0.78	0.77
[127]	2019	Team howard beale at SemEval-2019 task 4: Hyperpartisan news detection with BERT	Mutlu, O.	Semeval-2019	English	U.S.	DL	BERT	0.78	0.76
[126]	2019	Harvey mudd college at SemEval-2019 task 4: The clint buchanan hyperpartisan news detector	Drissi, M.	Semeval-2019	English	U.S.	DL	BERT	0.77	0.75
[90]	2019	Team ned leeds at SemEval-2019 task 4: Exploring language indicators of hyperpartisan reporting	Stevanoski, B.	Semeval-2019	English	U.S.	ML	Random Forest	0.77	0.74
[137]	2019	Team yeon-zi at SemEval-2019 task 4: Hyperpartisan news detection by de-noising weakly-labeled data	Lee, N.	Semeval-2019	English	U.S.	DL	BERT	0.76	0.76
[94]	2019	NLP@UIT at SemEval-2019 task 4: The paparazzo hyperpartisan news detector	Nguyen, D.V.	Semeval-2019	English	U.S.	ML	SVM	0.75	0.74
[40]	2019	Doris martin at SemEval-2019 task 4: Hyperpartisan news detection with generic semi-supervised features	Agerri, R.	Semeval-2019	English	U.S.	ML	Maxent	0.74	0.73
[74]	2019	Harvey mudd college at SemEval-2019 task 4: The carl kolchak hyperpartisan news detector	Chen, C.	Semeval-2019	English	U.S.	ML	Naive Bayes	0.74	0.74
[138]	2019	Steve martin at SemEval-2019 task 4: Ensemble learning model for detecting hyperpartisan news	Joo, Y.	Semeval-2019	English	U.S.	DL	CNN	0.74	0.70
[42]	2019	Cardiff university at SemEval-2019 task 4: Linguistic features for hyperpartisan news detection	Pórez-Almendros, C.	Semeval-2019	English	U.S.	DL	CNN + LSTM	0.74	0.71
[91]	2019	Team QCRI-MIT at SemEval-2019 task 4: Propaganda analysis meets hyperpartisan news detection	Saleh, A.	Semeval-2019	English	U.S.	ML	Logistic Regression	0.73	0.73
[89]	2019	Team fernando-pessa at SemEval-2019 task 4: Back to basics in hyperpartisan news detection	Cruz, A.	Semeval-2019	English	U.S.	ML	Random Forest	0.72	0.67
[108]	2019	Rouletabille at SemEval-2019 task 4: Neural network baseline for identification of hyperpartisan publishers	Moreno, J.G.	Semeval-2019	English	U.S.	DL	HAN	0.72	0.69
[45]	2019	Duluth at SemEval-2019 task 4: The pioquinto manterola hyperpartisan news detector	Sengupta, S.	Semeval-2019	English	U.S.	ML	Logistic Regression	0.70	0.68
[114]	2019	UBC-NLP at SemEval-2019 task 4: Hyperpartisan news detection with attention-based bi-LSTMs	Zhang, C.	Semeval-2019	English	U.S.	DL	LSTM	0.68	0.63
[104]	2019	Team xenophilius lovegood at SemEval-2019 task 4: Hyperpartisanship classification using convolutional neural networks	Zehe, A.	Semeval-2019	English	U.S.	DL	CNN	0.67	0.74
[47]	2019	Harvey mudd college at SemEval-2019 task 4: The d.x. beaumont hyperpartisan news detector	Amason, E.	Semeval-2019	English	U.S.	ML	Naive Bayes	0.65	0.73
[123]	2019	Team jack ryder at SemEval-2019 task 4: Using BERT representations for detecting hyperpartisan news	Shaprin, D.	Semeval-2019	English	U.S.	DL	BERT	0.64	0.64
[76]	2019	Team kermit-the-frog at SemEval-2019 task 4: Bias detection through sentiment analysis and simple linguistic features	Anthonio, T.	Semeval-2019	English	U.S.	ML	SVM	0.62	0.69
[103]	2019	Team peter brinkmann at SemEval-2019 task 4: Detecting biased news articles using convolutional neural networks	Fórber, M.	Semeval-2019	English	U.S.	DL	CNN	0.60	0.70
[113]	2019	Team kit kittredge at SemEval-2019 task 4: LSTM voting system	Cramerus, R.	Semeval-2019	English	U.S.	DL	LSTM	0.58	0.68
[131]	2019	Team harry friberg at SemEval-2019 task 4: Identifying hyperpartisan news through editorially defined metatopics	Afsarmanesh, N.	Semeval-2019	English	U.S.	Other	Gagavai Explorer	0.56	0.68
[73]	2019	Multi-task ordinal regression for jointly predicting the trustworthiness and the leading political ideology of news media	Baly, R.	MBFC	English	U.S.	ML	Copula Ordinal Regression	U/A	U/A
[85]	2019	Clark kent at SemEval-2019 task 4: Stylometric insights into hyperpartisan news detection	Gupta, V.	Semeval-2019	English	U.S.	ML	XGBoost	0.55	0.28
[139]	2019	Team peter-parker at SemEval-2019 task 4: BERT-based method in hyperpartisan news detection	Ning, Z.	Semeval-2019	English	U.S.	DL	BERT	0.50	0.61
[92]	2019	Tintin at SemEval-2019 task 4: Detecting hyperpartisan news article with only simple tokens	Bestgen, Y.	Semeval-2019 by-publisher	English	U.S.	ML	Logistic Regression	0.70	0.68
[65]	2019	Brenda starr at SemEval-2019 task 4: Hyperpartisan news detection	Papadopoulou, O.	Semeval-2019 by-publisher	English	U.S.	DL	CNN	0.66	0.70
[88]	2019	Fermi at SemEval-2019 task 4: The sarah-jane-smith hyperpartisan news detector	Chakravartula, N.	Semeval-2019 by-publisher	English	U.S.	ML	Random Forest	0.61	0.66
[60]	2019	Hyperpartisan news and articles detection using BERT and ELMo	Huang, G.K.W.	Semeval-2019 by-article + by-publisher	English	U.S.	DL	BERT	0.68	U/A
[58]	2019	Detecting political bias in news articles using headline attention	Gangula, R.R.R.	Telugu	Telugu	India	DL	HAN	0.89	U/A
[33]	2019	A stylometric inquiry into hyperpartisan and fake news	Potthast, M.	The BuzzFeed-Webis Fake News Corpus 2016	English	U.S.	ML	U/A	0.75	0.78
[70]	2019	Spider-jerusalem at SemEval-2019 task 4: Hyperpartisan news detection	Alabdulkarim, A.	Semeval-2019	English	U.S.	ML	SVM	0.74	0.71
[18]	2019	SemEval-2019 task 4: Hyperpartisan news detection	Kiesel, J.	Semeval-2019	English	U.S.	U/A	U/A	U/A	U/A
[142]	2019	NELA-GT-2018: A large multi-labelled news dataset for the study of misinformation in news articles	Nórregaard, Jeppe	NELA-2018	English	U.S.	U/A	U/A	U/A	U/A
[143]	2019	In plain sight: Media bias through the lens of factual reporting	Fan, L.	BASIL	English	U.S.	U/A	U/A	U/A	U/A
[148]	2019	Encoding social information with graph convolutional networks forPolitical perspective detection in news media	Li, C.	Allsides	English	U.S.	U/A	U/A	U/A	U/A
[69]	2018	Multi-view models for political ideology detection of news articles	Kulkarni, V.	Own	English	U.S.	Other	MVDAM	0.80	0.79
[44]	2018	Orwellian-times at SemEval-2019 task 4: A stylistic and content-based classifier	Knauth, J.	Semeval-2019	English	U.S.	ML	SVM	0.67	0.69
[144]	2018	Predicting factuality of reporting and bias of news media sources	Baly, R.	MBCF	English	U.S.	U/A	U/A	U/A	U/A
[145]	2018	Sampling the news producers: A large news and feature data set for the study of the complex media landscape	Horne, B.D.	NELA-2017	English	U.S.	U/A	U/A	U/A	U/A
[63]	2018	Understanding characteristics of biased sentences in news articles	Jeong Lim, S.	Own	English	U.S.	U/A	U/A	U/A	U/A
[52]	2015	Testing and comparing computational approaches for identifying the language of framing in political news	Baumer, E.	Own	English	U.S.	U/A	U/A	U/A	U/A

RQ1: Does a categorization for hyperpartisan news detection methods exist? Currently, there is no widely adopted comprehensive categorization system in the literature. The field still lacks standardized mathematical models for quantifying textual exaggerations that define hyperpartisan content. One key contribution of this systematic review is that it represents the first attempt to systematize news-based approaches while also enhancing the traditional PRISMA methodology by integrating ResearchRabbit during the "Identification of studies via other methods" phase. ResearchRabbit facilitated a systematic, data-driven expansion of our literature pool by visualizing clusters based on citation linkages. This clustering approach provided a structured method for identifying and selecting relevant studies by uncovering both direct citation relationships and keyword-based topic similarities. As a result, the tool contributed to a more comprehensive and cohesive expansion of the selected literature base. Furthermore, we proposed a specific definition of the studied phenomenon that can be applied in Computer Social Science and Computer Science.

RQ2: Is hyperpartisan news detection a stand-alone or overlapping task? The complexity of hyperpartisan news detection hints at an overlapping task encompassing various forms of media bias, suggesting a shift towards multi-label detection for nuanced representations. Research shows that models with fine-grained label sets outperform binary classifications, yet the majority of studies use simplified, binary categories.

RQ3: What are the proposed solutions using textual data? Research commonly applies text-based methods, such as Natural Language Processing (NLP) techniques, to detect hyperpartisan content by identifying linguistic patterns of exaggeration and emotional tone. In terms of labels, fine-grained labels show improved model accuracy in detecting diverse biases, but in this case the annotation required is costly.

RQ4: Does the task keep up with new NLP technologies like autoregressive models? To date, the adoption of advanced autoregressive models in hyperpartisan news detection is limited, revealing a critical gap. This gap underscores a need to explore these models, which could improve detection accuracy with state-of-the-art language understanding.

RQ5: What are the results of the developed models developed? Since the release of BERT, this model architecture—and particularly its variants, such as RoBERTa—has achieved state-of-the-art performance in a wide range of classification tasks.

RQ6: What datasets are used for this task? How are they structured? Have they been updated to cover the latest political global and regional trends? Datasets predominantly comprise English-language news articles, which risks skewing results when applying models to non-English contexts. Limited representation of minority languages restricts model generalization and hampers analysis of unique democratic and socio-political dynamics. In addition, dataset maintenance issues (e.g., broken URLs) hinder replicability, and paywalls or copyright constraints restrict access to time-sensitive data, impacting longitudinal research.

RQ7: How can the current state of research on hyperpartisan detection be characterized in diverse languages and countries? The absence of linguistically diverse datasets is a significant limitation, especially in minority and underrepresented cultures. This restricts the field’s capacity to develop effective hyperpartisan detection models for varied linguistic environments. Current datasets’ Anglo-American focus may limit models’ efficacy when applied to global democracies with different political and cultural contexts, exacerbating bias and misinformation issues in these areas. Moreover, the lack of cross-lingual studies leaves the impact of online environments on cultural-linguistic variations in hyperpartisan traits unexplored.

In conclusion, while existing research provides insights into hyperpartisan news, limitations in dataset diversity, language inclusion, and methodology highlight the need for more robust, globally representative resources. Future research could benefit from exploring autoregressive models and expanding cross-lingual analysis for a broader understanding of hyperpartisanship in diverse political systems and cultural contexts.
